# Prevalence and bidirectional association of sleep quality and gut health among Chinese midwives: a large population, multi-center cross-sectional study

**DOI:** 10.3389/fpubh.2024.1368178

**Published:** 2024-04-17

**Authors:** Jia-Ning Li, Qing-Xiang Zheng, Xiu-Min Jiang, Xiao-Qian Chen, Ling Huang, Yu-Qing Pan, Ru-Lin Liu, Yu Zhu

**Affiliations:** ^1^School of Nursing, Fujian Medical University, Fuzhou, China; ^2^Fujian Maternity and Child Health Hospital College of Clinical Medicine for Obstetrics & Gynecology and Pediatrics, Fujian Medical University, Fuzhou, China; ^3^Fujian Obstetrics and Gynecology Hospital, Fuzhou, China; ^4^School of Nursing, Fujian University of Traditional Chinese Medicine, Fuzhou, China

**Keywords:** gut health, midwives, nurses, sleep quality, shift work

## Abstract

**Background:**

Shift work can disrupt sleep quality and gut health. Nurses and midwives constitute approximately half of the global healthcare shift-working workforce. Our previous study revealed that most midwives were experiencing suboptimal health conditions, characterized by poor sleep quality and a high prevalence of gastrointestinal diseases. The gut–brain axis theory highlights the potential interplay between sleep quality and gut health. However, limited research focuses on this relationship among midwives.

**Methods:**

A cross-sectional survey included 2041 midwives from 87 Chinese hospitals between March and October 2023. Participants completed standardized questionnaires assessing sleep quality, gut health, depression, anxiety, and work stress. Binary logistic regression analyzed factors associated with poor sleep, and multiple linear regression examined the influence of sleep quality on gut health.

**Results:**

Over 60% of midwives reported poor sleep, with many experiencing gastrointestinal disorders. We observed a bidirectional relationship between sleep quality and gut health among midwives. After multivariable adjustments, midwives with higher gut health scores were more likely to experience poor sleep quality (odds ratio = 1.042, 95% confidence interval = 1.03–1.054). Conversely, midwives with higher sleep quality scores were also more likely to have poor gut health (*β* = 0.222, 95% confidence interval = 0.529–0.797). These associations remained robust across sensitivity analyses. Furthermore, depression, anxiety, and work stress significantly affected both sleep quality and gut health among midwives.

**Conclusion:**

This study enhances our understanding of the intricate relationship between sleep quality and gut health among midwives. Poor gut health was associated with a higher risk of poor sleep, and vice versa. To improve the overall wellbeing of midwives, the findings emphasize the importance of addressing poor sleep quality and promoting gut health through maintaining a healthy diet, lifestyle, and good mental health. Further studies are needed to confirm our findings and clarify the underlying mechanisms.

## 1 Introduction

Sleep quality among healthcare workers who rotate night shifts is a widespread concern (1). Nurses and midwives experience considerable declines in sleep quality due to their demanding roles (2). Midwives play indispensable roles in skilled birth attendance, emergency obstetric care, and immediate care for newborns. These duties, coupled with irregular working hours and emotional stressors, significantly impact their sleep quality (3). Their poor sleep can directly or indirectly impact the quality of midwifery care and maternal and infant health. However, there remains limited evidence regarding the sleep quality of midwives. Thus, exploring the sleep quality among midwives is crucial for addressing their wellbeing and job performance.

Nurses and midwives constitute a significant portion of the shift-working healthcare workforce and are susceptible to various health disorders associated with shift work (4, 5). Shift work acts as both a psychological and physiological stressor, increasing the risk of depression and anxiety (6), and disrupting sleep quality and gut health (7, 8). Gastrointestinal complaints are very prevalent among shift workers, affecting up to 81.9% of them (9), with no exception among nurses and midwives (10–12). While some studies have indicated that shift work may impact the gastrointestinal diseases of nurses (8, 9), it remains debated whether this effect is related to sleep quality, as some studies suggest that this association may be independent of sleep quality (11). Concurrently, several studies have highlighted the significant impact of sleep quality on adverse gut conditions (13–15). Understanding the impact of sleep quality on adverse gut conditions is crucial, especially given the emerging interest in the gut–brain axis theory.

The gut–brain axis theory proposes a bidirectional communication pathway between the gut and brain, through which humans regulate intestinal homeostasis and central nervous system function via neural networks and neuroendocrine, immune, and inflammatory pathways (16). The gut–brain axis has been observed in people with sleep disorders, where their gut health has also been influenced (17). This theory provides a framework for understanding the interconnectedness of sleep quality and gut health among midwives, who encounter unique challenges in maintaining both. Therefore, exploring the potential connections between sleep quality and gut health among them is essential.

Previous research has revealed that most midwives are experiencing suboptimal health conditions, characterized by poor sleep quality and a high prevalence of gastrointestinal diseases (12, 18). However, the relationship between sleep quality and gut health among midwives remains unexplored. Therefore, this study aims to examine the relationship between sleep quality and gut health among midwives in a large population. Based on the gut–brain axis theory, this study hypothesizes a bidirectional relationship between the sleep quality and gut health of midwives. Specifically, poor sleep quality may have a detrimental effect on gut health, while gut health may also influence sleep quality. Validating this bidirectional relationship will provide novel insights into improving the sleep quality and gut health of midwives, enhancing their overall wellbeing.

## 2 Methods

### 2.1 Study design, setting, and sample

This large population, multi-center, cross-sectional study aimed to assess the wellbeing of midwives working in obstetric wards and delivery rooms across a province. The survey was conducted from March to October 2023, using the Wenjuanxing online platform for convenience sampling. Hospitals conducting a minimum of 1,000 childbirths annually were chosen. In Fujian province, 102 eligible hospitals met this criterion, with 87 hospitals (85.29%) agreeing to participate.

Inclusion criteria for participation were as follows: (a) possession of professional qualification certificates and (b) willingness to participate in this study. Exclusion criteria were defined as follows: (a) retired nurses, refresher nurses, and nursing interns; (b) those with an employment duration of less than 6 months; and (c) those on extended leave for reasons such as illness, marriage, maternity, or other personal affairs exceeding 1 month. A total of 2,100 midwives participated, and 2041 were included for analysis after excluding cases with implausible data, such as unrealistic height, weight, body mass index (BMI), and survey responses with a duration of less than 300 s.

To ensure standardized data collection, research assistants were assigned in each city in Fujian and underwent online training sessions covering study objectives, participant inclusion and exclusion criteria, and data collection procedures. Research assistants then transferred responsibilities to nurse managers, who distributed the survey’s quick response (QR) code to eligible midwives. Participation was voluntary, and midwives could decline at any time. Each midwife had one opportunity to complete the survey, with all questions required for submission.

### 2.2 Survey questionnaires

#### 2.2.1 Demographic characteristics

We collected demographic data, including age, height, weight, educational level, marital status, number of children, monthly income, professional rank, employment type, hospital rank, hospital nature, department, traumatic childbirth experiences, weekly working hours, shift work, midday napping, exercise frequency, and shift work sleep disorder (SWSD). SWSD is a circadian rhythm sleep disorder characterized by complaints of insomnia and/or excessive sleepiness that are related to work schedules that occur during the usual sleep period. To assess SWSD, participants were asked three questions based on the International Classification of Sleep Disorders (ICSD-3) criteria: (a) Do you have a work schedule that sometimes overlaps with your usual sleep schedule? (b) If so, does this cause insomnia and/or excessive sleepiness due to reduced sleep amount? (c) If so, has this lasted for at least 3 months? Participants who answered “yes” to all three questions were classified as having SWSD.

#### 2.2.2 Sleep quality

Midwives’ sleep quality over the past month was assessed by the 19-item Pittsburgh Sleep Quality Index (PSQI) (19). The PSQI is a standardized self-rated questionnaire that generates a global sleep quality score from seven components: subjective sleep quality, sleep latency, sleep duration, sleep efficiency, sleep disturbance, sleep medication, and daytime dysfunction. Each item corresponds to a point value (0, 1, 2, or 3 points). The global PSQI scores range from 0 to 21, with higher scores indicating poorer sleep quality. Both the original English version and Chinese translation of the PSQI have undergone extensive validation, demonstrating excellent psychometric properties (19, 20). The Chinese version exhibits high internal consistency (Cronbach’s α = 0.82–0.83) and good test–retest reliability (*r* = 0.77–0.85) (20). A global PSQI score threshold of 7 is a diagnostic criterion in our study. This cutoff has a diagnostic sensitivity of 98.3% and a specificity of 90.2% for distinguishing good sleep from poor sleep in the Chinese population (21). In our study, Cronbach’s α was 0.743.

#### 2.2.3 Gut health

Gut health was assessed using the Gut–Brain Health Questionnaire (GBHQ), developed by the Institute of Psychology, Chinese Academy of Sciences (22). This questionnaire consists of 21 items divided into three dimensions: intestinal status (11 items), eating habits (6 items), and defecation status (4 items). Participants rate these items on a 5-point scale, with higher scores indicating worse gut health. The GBHQ has demonstrated good internal consistency (Cronbach’s *α* = 0.64–0.79) and reasonable test–retest reliability (*r* = 0.70–0.78) (22). In our study, Cronbach’s *α* was 0.807.

#### 2.2.4 Psychometric scales for depression, and anxiety

Depression symptoms were assessed by the Patient Health Questionnaire Depression Scale (PHQ-9), which was developed to identify depressive symptoms based on nine items (23). Each item is rated using a 4-point Likert scale (range: 0–3). The total score ranges from 0 to 27, with higher scores indicating more pronounced depressive symptoms. The Chinese version of the PHQ-9 has been shown to have good reliability (Cronbach’s *α* = 0.86) (24). In this study, Cronbach’s *α* was 0.911.

Anxiety symptoms were estimated by the generalized anxiety disorder (GAD-7), which was developed to identify anxiety symptoms based on seven items (25). Similar to the PHQ-9, each item is rated using a 4-point Likert scale (range: 0–3). The total score ranges from 0 to 21, with higher scores indicating more pronounced anxiety symptoms. The Chinese version of the GAD-7 has demonstrated good reliability (Cronbach’s α = 0.898) (26). In our study, Cronbach’s α was 0.936.

#### 2.2.5 Work start

Work stress was assessed using the Challenge- and Hindrance-Related Self-Reported Stress Measures (CHSS) (27). The CHSS consists of 11 items, including 6 items of challenge stressor and 5 items of hindrance stressor. Respondents rated each item on a 5-point Likert scale, ranging from 1 (no pressure) to 5 (extreme stress). The total score on the CHSS ranges from 11 to 55, with higher scores indicating higher levels of work-related stress. Cavanaugh et al. originally developed the CHSS, demonstrating commendable psychometric attributes for the scale, which encompassed satisfactory internal consistency of two subscale scores (Cronbach’s α ranged from 0.75 to 0.87) and discriminant validity (r = 0.28) (27). Additionally, Chinese scholars have translated it into Chinese and proved its applicability to Chinese operating theater nurses (28). In this study, Cronbach’s α was 0.913.

### 2.3 Statistical analysis

All statistical analyses in this study were conducted using IBM SPSS version 27. Baseline characteristics were provided as mean (standard deviation, SD) for continuous variables and frequencies (%) for categorical variables. Continuous variables were compared using t-tests or ANOVA; categorical variables were analyzed using chi-square tests; and Pearson’s correlation coefficient was used to estimate correlations. The odds ratios (ORs) and 95% confidence intervals (CIs) were estimated for independent variables in regression analyses. Crude ORs and 95% CIs were calculated in unadjusted regression models initially. Then, potential confounding factors were adjusted in the adjusted model. Binary logistic regression analyses were performed to investigate the influencing factors of poor sleep. To test the robustness of the results, we also performed sensitivity analyses, including adjustments for SWSD, the interaction term of weekly working hours × shift work group, and the exclusion of non-shift workers (*n* = 405) to rule out the effects of day shifts. Additionally, multiple linear regression analyses were conducted to further explore the influencing factors of gut health. Sensitivity analyses were also performed to test the results’ robustness. We adjusted the consumption of probiotics and gastrointestinal disorders, further adjusting for the weekly working hours × shift work group interaction term, as well as removing non-shift workers (*n* = 405). A *p*-value of 0.05 or less was considered statistically significant.

### 2.4 Ethical approval

This study received approval from the ethics committee of the main researcher’s hospital (No. 2022YJ071). Participants participated in the study voluntarily and had the option to withdraw their participation at any time. All methods in this study followed the relevant guidelines and regulations governing ethical research practices. All data are research data, and no participant could be identified.

## 3 Results

### 3.1 Characteristics of midwives

A total of 2041 midwives were included in this study, and the effective response rate was 97.19%. The predominant age group of midwives was 31–35 years, comprising 36.06%. Most midwives were married (78%) and had children (72.27%). Approximately half of the midwives had junior college degrees. A total of 45.32% of them fell within the income bracket of 3,000 to 5,999 RMB. Hospital types were well-distributed, with 54.24% from tertiary hospitals and 45.76% from secondary hospitals. Additionally, 1,023 (50.12%) midwives were working in the labor room and 1,018 (49.88%) in the maternity wards, respectively. The characteristics of midwives are presented in Table 1.

**Table 1:** Participant characteristics (*n* = 2,041).

Variable	Overall	Good sleep (*n* = 814)	Poor sleep (*n* = 1,227)	χ^2^ (*p*)	Gut health (*n* = 2,041)	t/F (*p*)
Age (year)						
≤25	214 (10.49)	96 (11.79)	118 (9.62)	6.281	53.77 ± 9.1	6.938
26 ~ 30	513 (25.13)	195 (23.96)	318 (25.92)	(0.179)	52.48 ± 10.19	(<0.001)
31 ~ 35	736 (36.06)	282 (34.64)	454 (37)		51.28 ± 11.31	
36 ~ 40	273 (13.38)	106 (13.02)	167 (13.61)		50.5 ± 10.98	
≥41	305 (14.94)	135 (16.58)	170 (13.85)		49.41 ± 11.39	
BMI						
Thin	206 (10.09)	91 (11.18)	115 (9.37)	2.468	52.6 ± 10.94	1.292
Normal	1,417 (69.43)	565 (69.41)	852 (69.44)	(0.481)	51.46 ± 10.9	(0.276)
Overweight	363 (17.79)	136 (16.71)	227 (18.5)		51.04 ± 10.51	
Obesity	55 (2.69)	22 (2.7)	33 (2.69)		49.96 ± 11.48	
Educational level						
Technical secondary school degree	70 (3.43)	32 (3.93)	38 (3.1)	1.090	52.23 ± 10.03	0.946
Junior college degree	1,014 (49.68)	405 (49.75)	609 (49.63)	(0.580)	51.72 ± 11.03	(0.388)
Bachelor degree and above	957 (46.89)	377 (46.31)	580 (47.27)		51.12 ± 10.72	
Marital status						
Unmarried	449 (22)	170 (20.88)	279 (22.74)	0.980	54.36 ± 9.48	7.071
Married	1,592 (78)	644 (79.12)	948 (77.26)	(0.322)	50.64 ± 11.08	(<0.001)
Number of children						
0	566 (27.73)	216 (26.54)	350 (28.52)	3.063	53.99 ± 9.96	15.480
1	727 (35.62)	282 (34.64)	445 (36.27)	(0.382)	50.91 ± 11.14	(<0.001)
2	727 (35.62)	306 (37.59)	421 (34.31)		50.13 ± 10.91	
≥3	21 (1.03)	10 (1.23)	11 (0.9)		48.24 ± 11.18	
Monthly income (RMB)						
<3,000	85 (4.16)	26 (3.19)	59 (4.81)	8.454	54.81 ± 12.32	4.337
3,000 ~ 5,999	925 (45.32)	361 (44.35)	564 (45.97)	(0.076)	51.59 ± 11.03	(0.002)
6,000 ~ 8,999	687 (33.66)	272 (33.42)	415 (33.82)		51.34 ± 11	
9,000 ~ 11,999	254 (12.44)	110 (13.51)	144 (11.74)		51.41 ± 9.5	
≥12,000	90 (4.41)	45 (5.53)	45 (3.67)		48.04 ± 9	
Professional rank						
Junior nurse	287 (14.06)	122 (14.99)	165 (13.45)	4.904	53.97 ± 10.16	7.789
Senior nurse	1,033 (50.61)	399 (49.02)	634 (51.67)	(0.179)	51.36 ± 10.69	(<0.001)
Assistant advanced nurse	616 (30.18)	242 (29.73)	374 (30.48)		50.9 ± 11.36	
Associate advanced nurses or Advanced nurses	105 (5.14)	51 (6.27)	54 (4.4)		48.84 ± 10.21	
Employment type						
Formal employees	815 (39.93)	340 (41.77)	475 (38.71)	1.906	51.31 ± 11.34	−0.507
Contract employees	1,226 (60.07)	474 (58.23)	752 (61.29)	(0.167)	51.56 ± 10.52	(0.612)
Hospital rank						
Tertiary	1,107 (54.24)	465 (57.13)	642 (52.32)	4.547	51.24 ± 10.51	−1.000
Secondary	934 (45.76)	349 (42.87)	585 (47.68)	(0.033)	51.72 ± 11.25	(0.317)
Hospital nature						
Specialized hospital	474 (23.22)	192 (23.59)	282 (22.98)	0.100	52.42 ± 10.14	2.316
General hospital	1,567 (76.78)	622 (76.41)	945 (77.02)	(0.752)	51.17 ± 11.05	(0.021)
Department						
Labor room	1,023 (50.12)	373 (45.82)	650 (52.97)	10.012	51.48 ± 10.64	0.097
Maternity wards	1,018 (49.88)	441 (54.18)	577 (47.03)	(0.002)	51.44 ± 11.07	(0.923)
Traumatic childbirth experiences						
No	1907 (94.94)	774 (95.09)	1,133 (92.34)	6.019	51.3 ± 10.79	−2.428
Yes	134 (6.57)	40 (4.91)	94 (7.66)	(0.014)	53.66 ± 11.58	(0.015)
Weekly working hours (hour)						
≤35	319 (15.63)	156 (19.16)	163 (13.28)	19.553	51.13 ± 10.65	2.036
36–40	1,158 (56.74)	465 (57.13)	693 (56.48)	(<0.001)	51.1 ± 10.96	(0.107)
41–45	475 (23.27)	158 (19.41)	317 (25.84)		52.52 ± 10.75	
≥46	89 (4.36)	35 (4.3)	54 (4.4)		51.61 ± 10.45	
Shift work						
None	405 (19.84)	195 (23.96)	210 (17.11)	14.398	49.44 ± 11.32	−4.199
Yes	1,636 (80.16)	619 (76.04)	1,017 (82.89)	(<0.001)	51.96 ± 10.68	(<0.001)
Midday napping						
None	75 (3.67)	22 (2.7)	53 (4.32)	33.681	52.88 ± 10.96	5.106
Rarely	220 (10.78)	67 (8.23)	153 (12.47)	(<0.001)	52.87 ± 10.45	(0.002)
Sometimes	745 (36.5)	264 (32.43)	481 (39.2)		52.14 ± 10.63	
Often	1,001 (49.04)	461 (56.63)	540 (44.01)		50.53 ± 11.03	
Exercise frequency						
None	811 (39.74)	296 (36.36)	515 (41.97)	23.325	52.16 ± 10.48	7.288
Less once a week	575 (28.17)	207 (25.43)	368 (29.99)	(0.001)	52.48 ± 11.04	(<0.001)
One to two times a week	438 (21.46)	209 (25.68)	229 (18.66)		49.85 ± 10.85	
Three times a week	105 (5.14)	50 (6.14)	55 (4.48)		50.65 ± 10.83	
More than three times a week	112 (5.49)	52 (6.39)	60 (4.89)		48.19 ± 11.36	

### 3.2 Status of sleep quality, gut health, depression, anxiety, and work stress

**Figure 1:**
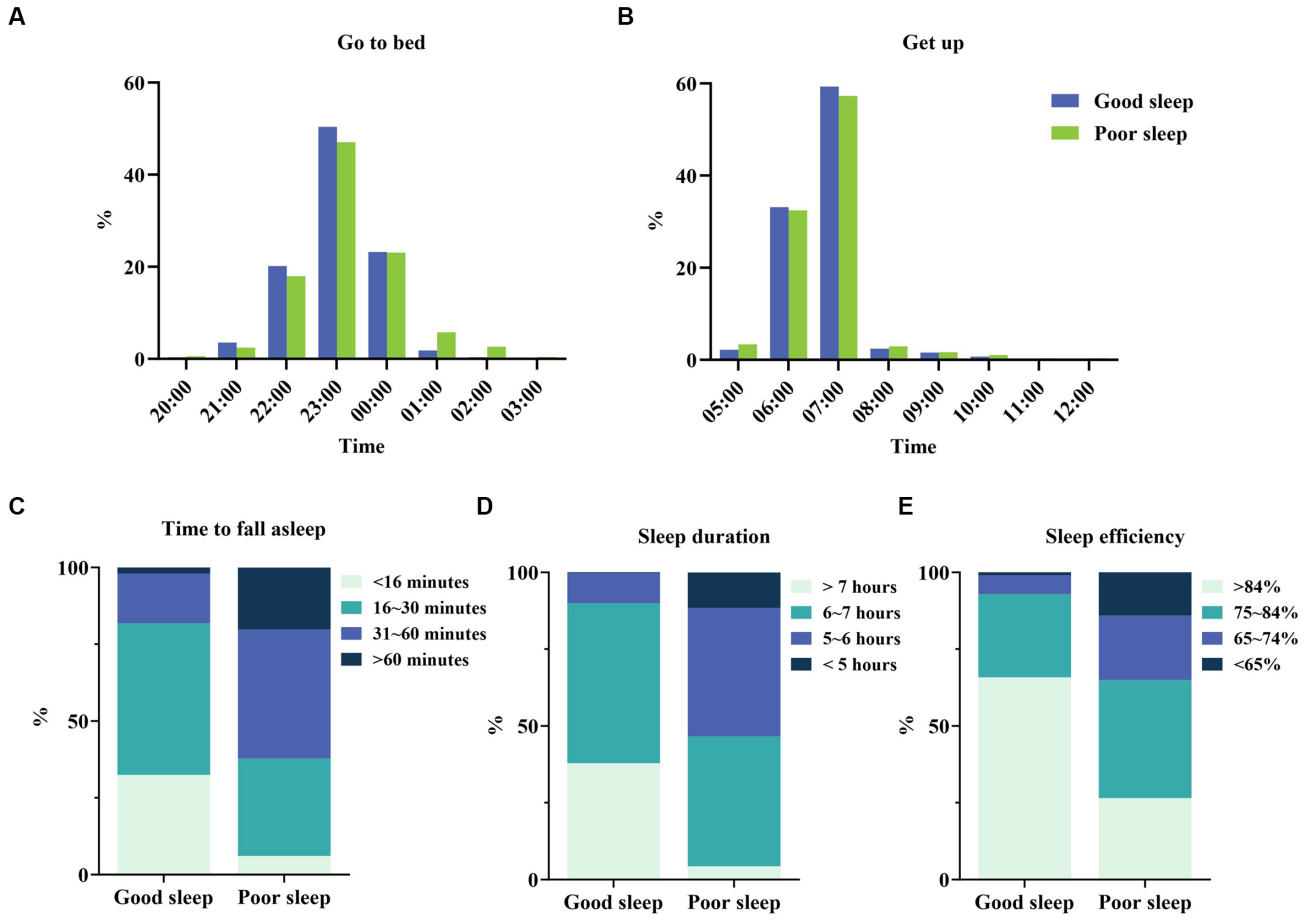
Sleep status in good and poor sleep groups. **(A)** Go to bedtime distribution for good sleep and poor sleep groups. **(B)** Get up time distribution on good sleep and poor sleep groups. **(C)** Difference in time to fall asleep between good sleep and poor sleep groups. **(D)** Difference in sleep duration between good sleep and poor sleep groups. **(E)** Difference in sleep efficiency between good sleep and poor sleep groups.

The PSQI score for sleep quality was 8.20(3.64). Detailed scores of the PSQI and its seven components are presented in Table 2. Among those, midwives scored poorer on the component for sleep medication and sleep efficiency. Besides, good and poor sleep quality were noted in 814 (39.88%) and 1,227 midwives (60.12%), respectively. The PSQI score for the good and poor sleep groups was 4.59(1.78) and 10.59(2.37), respectively (Table 2). The scores of PSQI and its seven components in the poor sleep group were higher than those in the good sleep group (*p* < 0.01). Additionally, most midwives reported going to bed between 22:00 and 24:00 and waking up between 6:00 and 7:00 (Figures 1A,B). Compared to the good sleep group, the poor sleep group had a longer sleep latency (Figure 1C). Furthermore, the poor sleep group had shorter sleep duration and lower sleep efficiency compared with the good sleep group (Figures 1D,E).

**Table 2:** PSQI scale score among midwives (*n* = 2,041).

Component	Overall	Good sleep (*n* = 814)	Poor sleep (*n* = 1,227)	t	*p*
Subjective sleep quality	1.51 ± 0.70	1.01 ± 0.47	1.85 ± 0.62	−34.68	<0.001
Sleep latency	1.7 ± 0.96	0.97 ± 0.72	2.18 ± 0.78	−35.91	<0.001
Sleep duration	1.26 ± 0.83	0.72 ± 0.64	1.61 ± 0.75	−28.49	<0.001
Sleep efficiency	0.91 ± 0.96	0.42 ± 0.65	1.23 ± 0.99	−22.05	<0.001
Sleep disturbance	1.21 ± 0.67	0.79 ± 0.5	1.48 ± 0.62	−27.83	<0.001
Sleep medication	0.13 ± 0.50	0.02 ± 0.17	0.21 ± 0.62	−10.24	<0.001
Daytime dysfunction	1.49 ± 1.05	0.66 ± 0.75	2.03 ± 0.84	−37.60	<0.001
PSQI global score	8.20 ± 3.64	4.59 ± 1.78	10.59 ± 2.37	−65.10	<0.001

**Figure 2:**
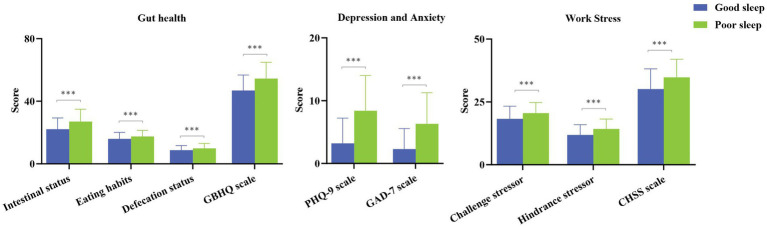
Gut health, psychological factors, and work stress in good and poor sleep groups. ***p < 0.001.

The GBHQ score for gut health was 51.46(10.85) (Table 3). Meanwhile, the scores of GBHQ and its three dimensions in the poor sleep group were higher than those in the good sleep group (Figure 2, *p* < 0.01). The PHQ-9 score for depression was 6.33(5.64), the GAD-7 score for anxiety was 4.70(4.80), and the CHSS score for work stress was 32.93(7.88) (Table 3). Plus, the scores of PHQ-9, GAD-7, and CHSS in the poor sleep group were higher than those in the good sleep group (Figure 2, *p* < 0.01).

**Table 3:** Correlations among the main variables (*n* = 2,041).

No.	Variable	Mean ± SD	Pearson’s correlation coefficients (*r*)			
			1	2	3	4
1	PSQI scale	8.20 ± 3.64	1			
2	GBHQ scale	51.46 ± 10.85	0.397^**^	1		
3	PHQ-9 scale	6.33 ± 5.64	0.512^**^	0.423^**^	1	
4	GAD-7 scale	4.70 ± 4.80	0.452^**^	0.407^**^	0.816^**^	1
5	CHSS scale	32.93 ± 7.88	0.333^**^	0.330^**^	0.474^**^	0.452^**^

**p < 0.01; ***p < 0.001.

### 3.3 Comparison of sleep quality, gut health, depression, anxiety, and work stress

We performed Pearson’s correlation analyses to further explore the correlation of sleep quality, gut health, depression, anxiety, and work stress (Table 3). The results showed that the sleep quality of midwives was positively correlated with gut health (*r* = 0.397, *p* < 0.01), depression (*r* = 0.512, *p* < 0.01), anxiety (*r* = 0.452, *p* < 0.01), and work stress (*r* = 0.333, *p* < 0.01).

### 3.4 Binary logistic regression analyses for poor sleep

Binary logistic regression analyses were conducted to investigate the relationship between gut health and sleep quality. In the unadjusted model, gut health was positively associated with sleep quality (OR = 1.077, 95% CI [1.066, 1.088], Table 4). When adjusting for shift work, both gut health and shift work were significant factors associated with poor sleep (OR = 1.076, 95% CI [1.065, 1.087]; OR = 1.327, 95% CI [1.050, 1.678], respectively, Table 4). We further adjusted for depression, anxiety, and work stress, and found that midwives with higher gut health scores were still more likely to have poor sleep quality (OR = 1.041, 95% CI [1.03, 1.053], Table 4). Based on the above results, the model was further adjusted for gut health, shift work, depression, anxiety, work stress, hospital rank, department, weekly working hours, midday napping, exercise frequency, and traumatic childbirth experiences. It illustrated that gut health was still an independent influential factor for poor sleep (OR = 1.042, 95% CI [1.03, 1.054], Table 4).

**Table 4:** Binary logistic regression for poor sleep.

Model	Gut health OR (95% CI)	Shift work OR (95% CI)	Depression OR (95% CI)	Anxiety OR (95% CI)	Work stress OR (95% CI)
Unadjusted	1.077 (1.066–1.088)***	1.526 (1.226–1.899)***	1.285 (1.251–1.32)***	1.29 (1.253–1.328)***	1.087 (1.072–1.101)***
Shift work adjusted	1.076 (1.065–1.087)***	1.327 (1.05–1.678)*	–	–	–
Shift work, depression, anxiety, and work stress adjusted	1.041 (1.03–1.053)***	1.21 (0.938–1.563)	1.178 (1.135–1.223)***	1.067 (1.025–1.112)**	1.022 (1.006–1.038)**
Multivariable adjusted^a^	1.042 (1.03–1.054)***	1.005 (0.765–1.321)	1.183 (1.139–1.229)***	1.069 (1.025–1.114)**	1.019 (1.002–1.035)*
Sensitivity analysis					
Further adjusted for SWSD	1.041 (1.029–1.053)***	0.784 (0.592–1.039)	1.168 (1.124–1.214)***	1.07 (1.026–1.116)**	1.011 (0.995–1.028)
Further adjusted for interaction effect (Weekly working hours × Shift work)	1.042 (1.03–1.055)***	0.637 (0.348–1.166)	1.18 (1.136–1.226)***	1.071 (1.027–1.116)**	1.019 (1.003–1.036)*
Excluded non-shift work	1.038 (1.024–1.051)***	–	1.184 (1.134–1.236)***	1.069 (1.018–1.123)**	1.022 (1.003–1.04)*

*p < 0.05; *p < 0.01; ***p < 0.001.

a Adjusted for gut health, shift work, depression, anxiety, work stress, hospital rank, department, weekly working hours, midday napping, exercise frequency, and traumatic childbirth experiences.

The occurrence rate of SWSD is a crucial variable linked to sleep quality among shift workers. So, we conducted further analysis to explore the rates of sleep quality and SWSD (Supplementary Figure S1). The result showed that 27.09% of midwives concurrently experienced SWSD and poor sleep, and the rates of SWSD between good and poor sleep quality groups were 16.6% and 45.1%, respectively, significantly different (*p* < 0.001) (Supplementary Figure S1). To test the result robustness, we also performed sensitivity analyses. The sensitivity results showed that it did not change substantially after adding the variable of SWSD (OR = 1.041, 95% CI [1.029, 1.053], Table 4), or additional adjustment for weekly working hours × shift work schedule group interaction term (OR = 1.042, 95% CI [1.03, 1.055], Table 4), or excluded non-shift workers (OR = 1.038, 95% CI [1.024, 1.051], Table 4).

### 3.5 Multiple linear regression analyses for gut health

Multiple linear regression analyses were conducted to further explore the relationship between sleep quality and gut health. In the unadjusted model, midwives with higher sleep quality scores were significantly more likely to have poor gut health (*β* = 0.397, 95% CI [1.065, 1.303], Table 5). After adjusting for shift work, both sleep quality and shift work remained significant predictors of gut health (*β* = 0.393, 95% CI [1.052, 1.29]; *β* = 0.067, 95% CI [0.73, 2.899], respectively, Table 5). We also further adjusted depression, anxiety, and work stress, midwives’ sleep quality was positively associated with gut health (*β* = 0.221, 95% CI [0.525, 0.79], Table 5). Moreover, in the multivariable-adjusted model, we included more various factors for adjustment, including sleep quality, shift work, depression, anxiety, work stress, hospital nature, age, professional rank, marital status, monthly income, number of children, midday napping, exercise frequency, and traumatic childbirth experiences. The results illustrated that midwives with higher sleep quality scores (poorer sleep quality) remained more likely to have poor gut health (*β* = 0.222, 95% CI [0.529, 0.797], Table 5).

**Table 5:** Multiple linear regression for gut health.

Model	Sleep quality *β* (95% CI)	Shift work *β* (95% CI)	Depression *β* (95% CI)	Anxiety *β* (95% CI)	Work stress *β* (95% CI)
Unadjusted	0.397 (1.065–1.303)***	0.093 (1.342–3.696)***	0.423 (0.739–0.89)***	0.407 (0.83–1.01)***	0.33 (0.399–0.512)***
Shift work adjusted	0.393 (1.052–1.29)***	0.067 (0.73–2.899)**	–	–	–
Shift work, depression, anxiety, and work stress adjusted	0.221 (0.525–0.79)***	0.056 (0.503–2.569)**	0.129 (0.115–0.381)***	0.138 (0.163–0.461)***	0.134 (0.124–0.244)***
Multivariable adjusted^a^	0.222 (0.529–0.797)***	0.021 (−0.597–1.734)	0.109 (0.076–0.343)**	0.141 (0.17–0.467)***	0.141 (0.134–0.255)***
Sensitivity analysis					
Further adjusted for consumption of probiotic and gastrointestinal disorders	0.17 (0.374–0.638)***	0.019 (−0.622–1.634)	0.069 (0.003–0.263)*	0.14 (0.173–0.462)***	0.141 (0.136–0.253)***
Further adjusted for interaction effect (Weekly working hours × Shift work)	0.225 (0.538–0.805)***	0.064 (0.135–3.371)*	0.112 (0.082–0.349)**	0.14 (0.168–0.465)***	0.147 (0.142–0.263)***
Excluded non-shift work	0.2 (0.448–0.756)***	–	0.091 (0.02–0.317)*	0.152 (0.168–0.503)***	0.136 (0.115–0.249)***

*p<0.05; *p < 0.01; ***p < 0.001.

a Adjusted for sleep quality, shift work, depression, anxiety, work stress, hospital nature, age, professional rank, marital status, monthly income, number of children, midday napping, exercise frequency, and traumatic childbirth experiences.

In the dynamic field of gastrointestinal health, the diversity and complexity of gut health cannot be overlooked. Therefore, we performed further analysis to obtain the rates of probiotic consumption and gastrointestinal diseases (Supplementary Figure S2). It was observed that 12.84% of midwives had consumed probiotics (Supplementary Figure S2A). Besides, among several common gastrointestinal disorders, the highest prevalence was associated with abdominal pain (35.57%), followed by constipation (28.42%), and functional dyspepsia (23.86%) (Supplementary Figure S2A). When it comes to each of these gastrointestinal disorders, the poor sleep group exhibited a higher prevalence compared to those in the good sleep group (*p* < 0.01) (Supplementary Figures S2B,C).

Sensitivity analyses were also conducted to assess the robustness of our findings. After adjusting for the consumption of probiotics and gastrointestinal disorders, the association also persisted (*β* = 0.17, 95% CI [0.374, 0.638], Table 5). Meanwhile, when we considered the interaction effects of weekly working hours × shift work schedule group or excluded daytime workers, the results remained consistent (*β* = 0.225, 95% CI [0.538, 0.805]; *β* = 0.2, 95% CI [0.448, 0.756], respectively, Table 5).

## 4 Discussion

Overall, the study aimed to explore the association between sleep quality and gut health among Chinese midwives according to the gut–brain axis. This study found that more than half of midwives experienced poor sleep, and they also suffered from gastrointestinal disorders such as abdominal pain, constipation, and functional dyspepsia. The primary findings indicated a bidirectional relationship between sleep quality and gut health. Poor sleep adversely affected gut health, and conversely, gut health influenced sleep quality, supporting the hypothesis of this study. Furthermore, depression, anxiety, and work stress significantly impacted both sleep quality and gut health among midwives.

Our study confirms the prevalence of poor sleep among midwives, reaching a staggering 60.12%, surpassing rates observed in previous research on nurses and midwives (2). Notably, 27.09% of midwives concurrently experienced SWSD and poor sleep. This heightened prevalence may stem from the pivotal role midwives play in childbirth and maternal care, particularly due to the implementation of the three-child policy by the Chinese government, which encourages couples to have a third child (29). The additional demands and responsibilities resulting from this policy change have further exacerbated the issue of sleep quality among midwives.

Our study demonstrated that the gut health among midwives was suboptimal, and midwives suffered many gastrointestinal disorders such as abdominal pain, constipation, functional dyspepsia, and so on. The inherent emotional demands of the midwifery profession can lead to chronic stress, which may disrupt the regular operations of their digestive systems. Additionally, irregular schedules pose challenges for midwives in maintaining healthy habits, including regular eating. Poor lifestyle choices can further contribute to nutritional deficiencies and digestive disturbances, underscoring the significance of dietary choices in shaping gut health (30).

The gut–brain axis theory posits a bidirectional communication between the gut and brain (16), a concept that was confirmed in this study. Our finding observed a bidirectional relationship between sleep quality and gut health among midwives. Binary logistic regression analyses supported that midwives experiencing gut health concerns were more likely to suffer from poor sleep, a finding consistent with previous studies on patients with gastrointestinal diseases (31, 32). A healthy gut plays a crucial role in regulating sleep, as it produces various sleep-regulating compounds, including neurotransmitters such as serotonin, which help maintain normal sleep quality (33). Moreover, gut health is intricately linked to the immune system and inflammation. Chronic inflammation in the gut can trigger a systemic inflammatory response, which has been associated with the development of sleep disorders (34). Inflammatory molecules can interfere with the sleep–wake cycle and disrupt the circadian rhythm, leading to poor sleep quality. Additionally, disruptions in gut health often manifest as abdominal discomfort and pain, further exacerbating difficulties in falling and staying asleep.

In turn, multiple linear regression analyses demonstrated that midwives reporting poor sleep quality were more likely to experience gut health concerns. This correlation aligns with previous research linking digestive symptoms and sleep disturbances (35). Additionally, Loosen et al. (36) indicated that sleep quality could serve as an indicator of gastrointestinal cancer. Healthy sleep patterns are essential for hormonal regulation. Disturbances in the sleep patterns of shift workers can lead to disturbances in the circadian rhythms of hormones (37). Consequently, poor sleep quality and hormonal imbalances may compromise immune and inflammatory responses, rendering the gut more susceptible to infections and inflammation (34). Emerging evidence suggests that sleep disturbances can affect the composition and diversity of gut bacteria (38), potentially leading to dysbiosis and increased susceptibility to gut health issues.

Another noteworthy finding from our study highlights the significant role of psychological factors, particularly depression and anxiety, in influencing both sleep quality and gut health. This finding is consistent with prior research (39, 40). Depression and anxiety are known to affect monoamine neurotransmitter levels, such as serotonin and norepinephrine, which are synthesized not only in the brain but also in the gut (41, 42). Furthermore, depression and anxiety often co-occur with sleep disorders such as insomnia, creating a vicious cycle where one condition exacerbates the other (43). Thus, the mental health issues of midwives need urgent attention. Future studies could consider the mediating role of psychological factors that nuance the bidirectional interplay between sleep quality and gut health.

Additionally, high work stress among midwives poses risks to both gut health and sleep quality. Work stress can induce a range of stress responses, potentially disrupting eating patterns and exacerbating gastrointestinal symptoms (44). Stressors in the work environment, such as urgent tasks and the unpredictability of patient outcomes, can affect sleep quality by triggering the release of stress hormones (45). Work stress is an inevitable aspect of the midwifery profession. Cultivating resilience to cope with work stress is crucial for midwives, as it can help them better manage the challenges of their profession, ultimately promoting improved sleep quality and gut health.

Consistent with previous studies (7, 46), shift work has been linked to changes in both gut health and sleep quality. These associations are believed to be influenced by disruptions in circadian rhythms, alterations in eating habits, and increased stress levels commonly experienced by those working shifts. However, it is unexpected that, after adjusting for various factors, the impact of shift work on gut health and sleep quality may diminish. This discrepancy may arise from the inclusion of a majority of shift workers and a smaller proportion of non-shift workers in the study, potentially leading to incomparability and an incomplete understanding of the true effects. Future research should aim to include larger and more diverse samples of both daytime and shift workers to comprehensively investigate the bidirectional relationship between sleep quality and gut health.

### 4.1 Strengths and limitations

Our study has several strengths. First, we collected a larger sample size to draw more reliable conclusions and provide new insights from gut health in the gap of midwives’ sleep-related management interventions. Second, we also adjusted for potential risk factors and performed sensitivity analyses to ensure robustness. However, several limitations to this study were also inevitable. First, despite conducting separate analyses of the impact of gut health on sleep quality, and the impact of sleep quality on gut health, the cross-sectional design could not allow inference of causal relationships between these variables. Second, the current explanatory variables in this study were exclusively self-reported, including sleep quality and gut health, even though all scales have been shown to be reliable. There was still a lack of data on objective testing, such as collecting stool specimens to observe gut microbiota by 16S rRNA sequencing and sleep data through non-invasive techniques such as actigraphy.

## 5 Conclusion

Our study reveals a bidirectional association between sleep quality and gut health among midwives. Poor gut health contributes to poor sleep, and conversely, poor sleep negatively impacts gut health. Additionally, factors such as depression, anxiety, and work stress significantly affect both sleep quality and gut health in this population. Recognizing the dual impact of sleep quality and gut health on midwives’ wellbeing, strategies should be implemented to enhance their overall health. These strategies may include dietary and lifestyle modifications, such as promoting healthier eating habits and incorporating stress management techniques. Additionally, brain-gut behavior therapies and microbiome-enhancing strategies can play a crucial role in improving the interconnected factors influencing sleep quality and gut health. By addressing these multifaceted factors, healthcare organizations can foster environments that prioritize the holistic wellbeing of midwives. Further research is warranted to clarify the underlying mechanisms driving these associations, which will inform the development of tailored interventions to support the health and vitality of midwives and healthcare professionals, thereby enhancing patient care and outcomes.

## Data availability statement

The raw data supporting the conclusions of this article will be made available by contacting with the corresponding author.

## Ethics statement

The studies involving humans were approved by Fujian Maternity and Health Hospital ethics committee of the main researcher’s hospital (No. 2022YJ071). The studies were conducted in accordance with the local legislation and institutional requirements. Participants participated in the study voluntarily and had the option to withdraw their participation at any time.

## Author contributions

J-NL and Q-XZ: Conceptualization, Data curation, Investigation, Methodology, Project administration, Formal analysis, Writing – review & editing, Writing – original draft. X-MJ: Conceptualization, Methodology, Project administration, Supervision, Resources Writing – original draft, Writing – review & editing. X-QC: Conceptualization, Methodology, Investigation, Writing – original draft. LH: Conceptualization, Validation, Writing – original draft. Y-QP: Data curation, Formal analysis, Investigation, Writing – original draft. R-LL: Data curation, Formal analysis, Methodology, Project administration. YZ: Formal analysis, Investigation, Writing – original draft.
