# Supplementary figures and images for: Prevalence and bidirectional association of sleep quality and gut health among Chinese midwives: a large population, multi-center cross-sectional study
Source: Front Public Health. 2024 Apr 17;12:1368178. doi: 10.3389/fpubh.2024.1368178 (PMC11061365; doi:10.3389/fpubh.2024.1368178)

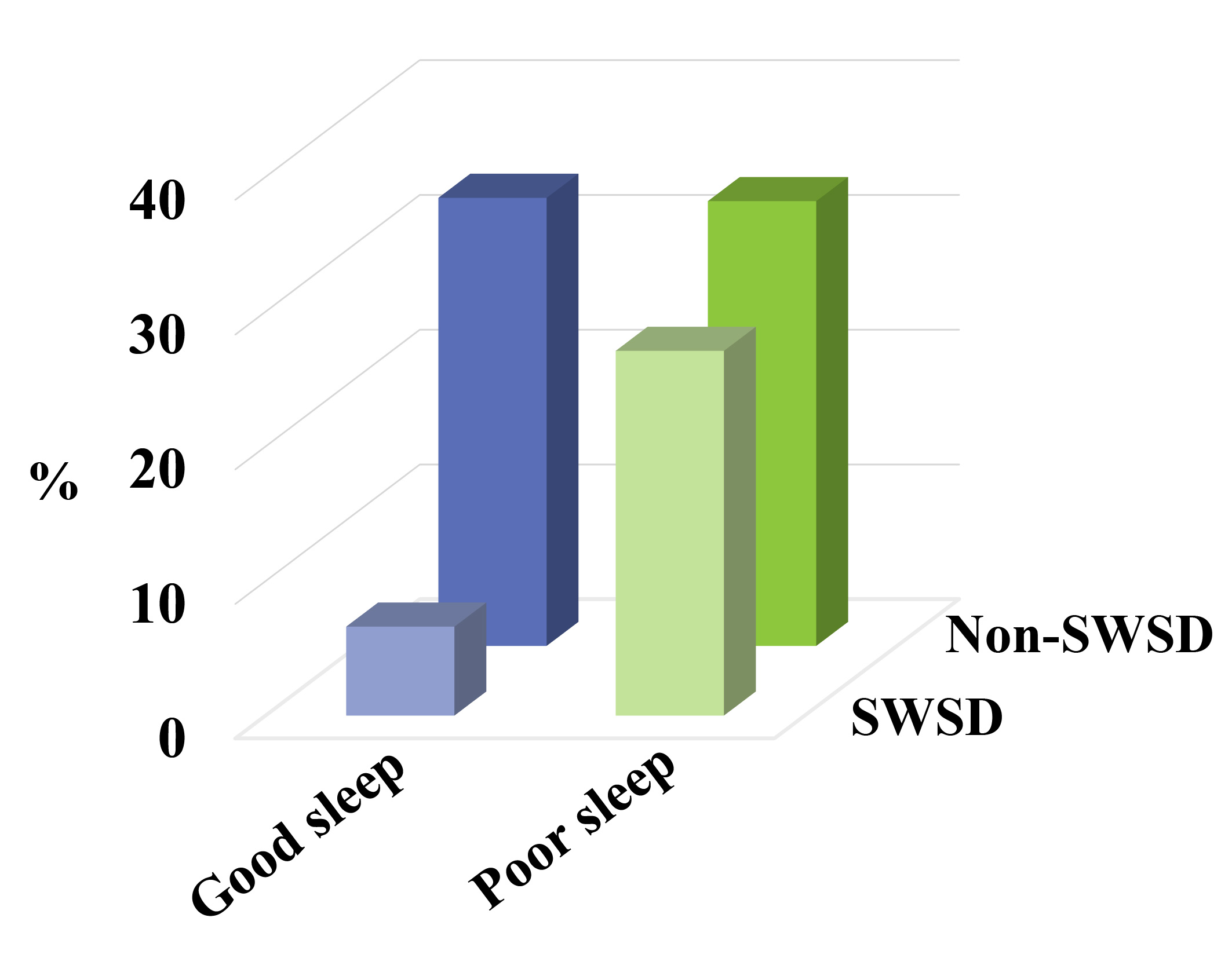

Supplement: Supplementary file 1 [file Image_1.JPEG]

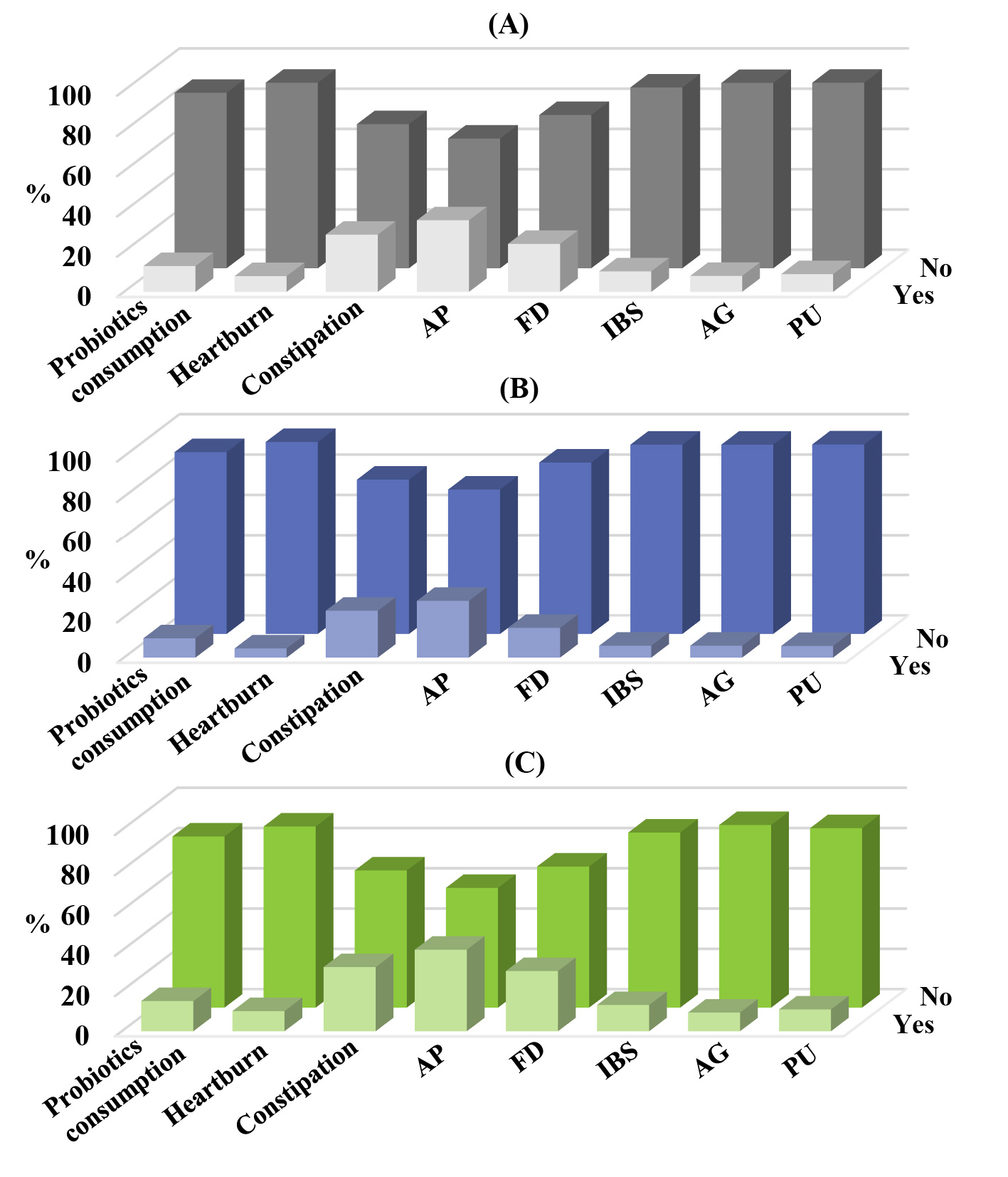

Supplement: Supplementary file 2 [file Image_2.JPEG]
